# Assessment of incentivizing effects for cancer care frameworks

**DOI:** 10.1007/s10585-020-10046-y

**Published:** 2020-06-17

**Authors:** Jörg Haier, Jonathan Sleeman, Jürgen Schäfers

**Affiliations:** 1 Comprehensive Cancer Center, Hannover Medical School, Hannover, Germany; 2 Medical Faculty Mannheim, University of Heidelberg, CBTM, Ludolf-Krehl-Str. 13 - 17, 68167 Mannheim, Germany; 3 Institut für Toxikologie Und Genetik, Karlsruhe Institute for Technology (KIT), Campus Nord, Postfach 3640, 76021 Karlsruhe, Germany; 4 IGP-Institute for Health Sciences and Public Health, Muenster, Germany

## Importance of incentives for cancer care

A central goal for healthcare systems worldwide is to provide sufficient cancer care to the population. However, it represents a major hurdle in many low- and middle-income countries (LMICs), resulting in an unnecessarily high proportion of patients with metastatic and advanced tumor stages. For many of these countries, significant differences between regions, between rural and urban populations, and across social groups remain major challenges for the reorganization of existing cancer care delivery, and the implementation of new structures that enable basic equal access to cancer care for the entire population. In our editorial series about incentives for cancer care in LMICs we have outlined how healthcare development in LMICs can be supported and guided by setting the right incentives. These encourage and steer the involved stakeholders towards improving the availability, acceptability, affordability and accessibility of sufficient quality of care, particularly for vulnerable populations such as those with metastatic disease. Financial as well as sociocultural incentives can motivate participants in the cancer care system (individuals, entities as well as organizations) to implement and carry out specific measures. However, the effects that are achieved are dependent on the healthcare framework. Setting incentives is frequently associated with potential conflicts of interests (CoI) that can potentially harm patients. When CoI and other forms of decisional bias are sufficiently excluded or controlled, the intentional use of various types of incentives can promote beneficial cancer care development [1]. These incentives are not limited to physicians, and can impact on many stakeholders and professions, even including patients themselves, their relatives, as well as healthcare politicians.

Key financial incentives, their specific attributes and their potential impacts are summarized in Table 1. The incentives include different financial models for reimbursement, such as fixed salaries, capitation, fee for service, or performance-based schemes, and can exert specific effects on cancer care systems. However, different targets within cancer care systems (for example the population covered by the cancer care system, risk selection, the number of treated cases, and the efficiency of resource usage) are influenced in different ways. Therefore, special attention needs to be paid when establishing effective incentive frameworks for the management of cancer patients. These incentive frameworks need to take into account the vulnerability of patients with advanced cancer, the often life-threatening course of their disease, as well as their dependence on costly diagnosis and treatment. In view of this, we suggest that for LMIC it may be beneficial to separate cancer care from other healthcare areas with regard to financial incentives and reimbursement schemes [2].

**Table 1:** Most important opportunities, strengths, threads and weaknesses of financing and reimbursement schemes with regard to LMIC

Incentive/payment	Opportunities	Strengths	Threads	Risks
Fixed salaries	High ability for cost forecast Motivation in underserved regions	Full availability of infrastructure for treatment	Low incentivizing effects (quality and quantity)	In the long run: incalculable system costs Avoidance of complex treatments
Capitation	Strengthening the referral aspects of the system Assurance of basic UHC provision to broad population	Motivating patient enrollment into insurance Stimulation of acceptance and availability of healthcare structures	Tends to induce to ineffective usage of capacities	Lacking to stimulate quality and efficiency Preference of patients with easy conditions and treatments
Case-related payment	Reimbursement is orientated towards national cost structures and healthcare priorities	Stimulus towards providers for quality in treatment Efficient usage of resources	Availability of reliable data and reporting structures	Exigent evaluation and controlling processes High economic pressure on the entire system Reimbursement driven patient selection
Procedure related payment	Output-related structure focusing on treatment System guidance towards prioritized medical approaches	Stimulates specialization and high case loads Establishes comparability of treatments and costs	Huge amount of data necessary to define comparable procedures Preference for procedures with high technological impact and infrastructure requirements	The expenditure-driven data is often not suitable to the quality assurance purposes Underfinancing and insufficient provision of treatment with low technical requests, preventive and narrative-based medicine
Integrated care	Multidisciplinary approaches High ability for cost forecast	Patient-centered treatment and service organization High impact on patient’s quality of life	Integration of high number of stakeholders in the patient-process High management efforts Low evidence in efficiency regarding to performance	Selection of the involved parties regard to the reimbursement Time-consuming barriers for implementation of innovations
Pay-for-performance (P4P)	Providers can be financially rewarded for providing high-quality and financially penalized for providing low-quality care	If incentives are valuable enough: stimulation of investment into improved care (infrastructure, training, management) Intensive political guidance function	Significant difference between the existing models Definition of reliable and measurable quality indicators High management, reporting and controlling efforts	Inadequate risk adjustment and accounting for differences in underlying patient populations Selection of patients with low complexity, comorbidities and other risk factors
Disease management programs (DMPs)	Assurance of cooperation between care seekers and care providers Supports implementation of treatment guidelines and standards	Using a mix of financial and non-financial instruments Clear definition of included benefits and expectations Establishes comparability of treatments and costs	Singular focus on financially incentivizing providers is unlikely to stimulate uptake Only suitable for small number of diseases or medical conditions with highly standardized and comparable medical approaches High management, reporting and controlling efforts	Mixed evidence about the quality improvement and cost savings potential of DMPs but financial incentives to sickness funds
Hospital value-based purchasing	Aimed to improved performance of single providers on a series of quality metrics	Transparency and comparability of treatment quality Stimulates specialization and high case loads Establishes comparability of treatments and costs	Providers are intensively motivated to avoid patients with clinically complex conditions or economic risks Low priority of long-term outcome aspects due to inability of integration into value definitions to be translated reimbursement	Without current evidence for improved patient outcome Economic effects depend from vary different healthcare frameworks Impairing cross-sectoral healthcare provision

The allocation of qualitatively and quantitatively sufficient human resources to cancer care in LIMCs demands that sociocultural incentives are also understood and strategically used [3]. Similar to financial incentives, factors that influence that motivation of cancer care providers are intensively related to successful establishment of delivery models and the removal of implementation barriers. The entire cancer care community including all professionals groups, providers, organizations and policy makers therefore needs to balance the various types of incentives for each category of participant in the cancer care system. Furthermore, in order to guide the use of incentives in a dynamic manner, their effects need to be regularly assessed.

## Assessment of incentivizing effects of cancer care framework

Incentivizing effects are always present in cancer care systems. They are heavily influenced by the political, economic, legal and sociocultural frameworks that are prevalent in each country. The resulting effects are dynamic, and differ between the various sociocultural environments. Therefore, due to rapidly changing societies, economic developments and prioritized efforts for Universal Health Coverage (UHC) in LMICs, awareness and consideration of incentivizing effects within the given healthcare framework is crucial not only for successful implementation but also for achievement of the expected outcomes. Awareness and anticipation of incentivizing effects need to be ensured before novel framework conditions are set in place. In addition, incentivizing effects need to be continuously evaluated and supervised during and after implementation of these changes. Evaluations have to cover the individual, organizational and systemic levels, and include all major stakeholders and UHC aspects. As far as possible, the performance of any cancer care system should be assessed with respect to the scope and quality of services actually received by patients.

Before implementation of incentivizing measures, such as financial incentives, reimbursement schemes and additional economic incentives, a number of parameters need to be carefully considered, and their potential and expected consequences need to be continuously assessed. For example, impacts on the covered population, the number of treated cases, and the number and complexity of procedures need to be estimated. Effects on risk selection, the accessibility of cancer care structures, and possible barriers for cancer care use should be appraised. In addition, the consequences for the clinical quality of cancer care and the efficient use of resources need to be gauged.

During and after implementation, there needs to be continuous supervision and assessment of the effects that are realized. To this end, predefined indicators need to be implemented to structure the ongoing measurement and quantification of the incentivizing effects.

Evaluation of the impact of sociocultural incentives needs to be carefully designed, as these may be a direct component of novel healthcare frameworks, but can also have indirect effects on cancer care delivery processes. Key areas for consideration include professional education (including supervision and training), career development, and professional migration within the system, both nationally (rural, remote, urban) and internationally (loss and/or gain of healthcare professionals and knowledge). In addition, a number of social aspects have important incentivizing effects that need to be monitored, including housing, volunteer motivation, social recognition and community appreciation. The motivational impact of sociocultural incentives provided by the available infrastructure (including human resource environment) needs to be evaluated, as do innovations and delivery processes for various healthcare professional groups that enable them to provide a high quality of care.

For all analytical steps it is necessary to predefine a catalogue of indicators that allow the evaluation parameters outlined above to be measured. These may differ between countries and healthcare systems, and can depend on priority definitions and cultural value systems. Indeed, the evaluation of incentivizing effects always need to take into account the general aim of UHC, and to identify potential misuse of these incentives as inappropriate CoI.

In summary, different types of incentives have varying effects on the successful implementation of cancer care in LMICs, which can be particularly decisive for the effective management of patients with metastatic disease. The results of incentives can be stimulating, but can also build up additional barriers for healthcare system development. Their impact is subject to constant change, and depends on the prevailing environmental framework. For all types of incentives, transparency, public discussion and strategic implementation should be considered as a prerequisite for achieving progress towards UHC, in particular for the implementation of full cancer care coverage for all populations in LMICs, and especially for those patients with metastatic disease.
